# Retrospective and Apologetic

**Published:** 1863-02

**Authors:** C. C. Dills


### RETROSPECTIVE AND APOLOGETIC.

BY C. C. DILLS, D. D. S.

Rivoral has it, that "Genius is only great patience;" and another has said, that a manuscript should be nine years old before its publication. These are valuable suggestions, and had I appreciated them six months ago, or more, the series of articles just closing would not now have been commenced. I have been chagrined all along, to see to what extent I was sacrificing good headings for articles, rather than to have been more patient in commencing them. But genius I had not, or, rather, that I had was overcome, by a request to write, and my ignoring the little favorable time in which I would have to do it. And again, I thought to encourage Rivoral's idea of genius, might be to engender sloth; which, certainly, to defer publishing, for nine years, articles on any subject of progressive dentistry, would be most seriously to endanger their utility. I will not apologize farther. I have written, first, for my own pleasure and profit; and, second, for that of others.

The first is commendable, as looking to self for benefit; the second, as looking to benefit outside of self—to discharge a duty and sustain a good cause. Really, I feel under positive obligation, not only *to think*, but to put such of my thoughts as are probably worth it *on record*, *for dentistry*. As vary the autumnal tints, so do our minds; and as it takes all of human thought and action, to constitute human life, so should be given all of each dentist's peculiar professional knowledge, to make perfect the dental profession. My share may be inferior enough, but I have this to console me in giving it, that the pages of a dental journal to-day, are scarcely as valuable as were those of the Alexandrian or Vatican manuscripts in their day; and, besides, it may be altogether proper for the *Register* that it have its department for local, novel, lighter or less important matter, as well as that for the most weighty truths, or the highest professional knowledge.

For my part, I no more want to see exclusive matter of fact in the *Register*, than I want exclusive contemplation of the *real* any where; no more want it all microscopic and chemical, than I want business monotinous and unattractive. I would not be misunderstood here; I would not detract from any one's effort to write the best he can, but I would have it encourage him to write at all. In this, too, the *Editors Register*, I trust, will find sufficient excuse for some liberty taken.

PIQUA, O., *January*, 1863.

*Further corrections.*—Insert the following sentence *immediately after* the illustration: "My lads," said Napoleon, etc., to-wit: "I like, when we must battle, the desperation of fatality—it is virtue and strength, then, where glory and victory are won."

*Still another, to-wit.*—Instead of this sentence—"If I talk with a man, but more especially a woman, whose mouth grows wide and face falls in," *put this:* "If I talk with a toothless man, but more especially with a toothless woman, I fully expect him to become my patient," etc.

